# Design of open systems for meniscus splitting demonstrated using an aqueous polymer solution

**DOI:** 10.1080/14686996.2025.2512704

**Published:** 2025-05-28

**Authors:** Reina Hagiwara, Kosuke Okeyoshi

**Affiliations:** Graduate School of Advanced Science and Technology, Japan Advanced Institute of Science and Technology, Ishikawa, Japan

**Keywords:** Humidity, evaporation rate, polyvinyl alcohol, meniscus splitting, dissipative structure

## Abstract

Open systems on the surfaces of soft materials induce dissipative structures such as evaporative self-organization. Based on the viscous fingering phenomena, we demonstrate a meniscus splitting phenomena by evaporating an aqueous solution of versatile polyvinyl alcohol (PVA) solution from a Hele–Shaw cell. To understand it as a universal phenomenon independent of the polymer species, the distinct evolutionary pathways to pattern formation were evaluated, focusing on the interface deformation and directional deposition of the polymer. The PVA solution, which showed a steep concentration-dependent viscosity gradient under a local moderate humidity gradient, was capable of bridging the cell gap for vertical membrane formation. The interfacial shape transformation from one downward concave to multiple upward convex shapes is controllable not only by tuning the physical properties of the polymer but also by conditioning the water evaporating atmosphere. We envision that demonstrations with different cell aperture designs will expand the realization of this phenomenon using other synthetic polymers or chemical species. Such an open system design with humidity tuning is a strategy for exposure to non-equilibrium phenomena using various soft materials, particles, fibers, and networks.

## IMPACT STATEMENT

‘Meniscus splitting’ is demonstrated by using polyvinyl alcohol solutions for highlighting universality in open systems. The interfacial convexity can be critically deformed upward or downward under control of humidity gradient.

## GRAPHICAL ABSTRACT

**Figure:**
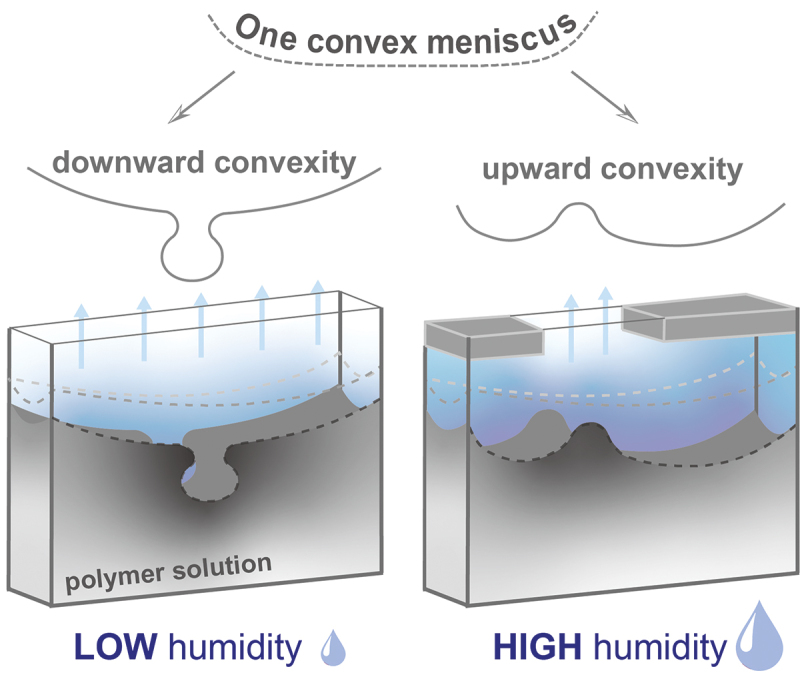

## Introduction

One of the dissipative structures [1], viscous fingering [2–4], is a well-known phenomenon showing that interfacial instability [5] on the surfaces of soft materials and geometrical deformation. When soft materials are in a fluid state in a drying environment, various physicochemical processes [6–8] such as flow [9–14], diffusion [15–17], phase separation [18,19], and gelation [20–23] occur in a non-equilibrium state. This mass influx/efflux contributes to the organized structure of the dried state, such as crystal growth. As one of the pattern formations resulting from the drying of viscous polymer solutions, we recently reported a ‘meniscus splitting phenomenon’ [24–27]. This characteristic time evolution of splitting one meniscus into multiple with spontaneous spatial patterns under non-equilibrium interfacial conditions during solvent evaporation, like drying-induced pattern formation [28–30]. This is an expansion of viscous fingering, changing the interface from an even shape to a wave shape [31–33]. When the solvent of the polymer solution evaporates in a cell with a gap of ~1 mm, the capillary forces acting on the evaporative interface cause the polymer to deposit by bridging the cell gap at multiple specific positions within the characteristic interval. Previously, the meniscus splitting phenomenon has been demonstrated with high reproducibility using several natural polysaccharides such as pectin [34,35] and chitosan [36]. Because polysaccharides exist in harmony with water in vivo, their drying and wetting interface is highly functional as they not only act as nutrient storage mediators in aqueous environments but also as moisturizers in dry-air environments.

However, the interfacial deformation processes immediately before meniscus splitting are assumed to follow solvent evaporation, and it is essential to prove the universality of this phenomenon by demonstrating its species independence. As the first step, we choose polyvinyl alcohol (PVA), a versatile synthetic polymer possessing biocompatible and biodegradable properties and has a high affinity for water. PVA is a representative hydrophilic synthetic polymer with a chemical structure containing many hydroxy groups. The strategy is to extend the chemical species having similar properties originating from functional groups, as previously demonstrated in polysaccharides. PVA has a simple polymeric structure and a different backbone to polysaccharides such as pectin.

**Figure 1.**
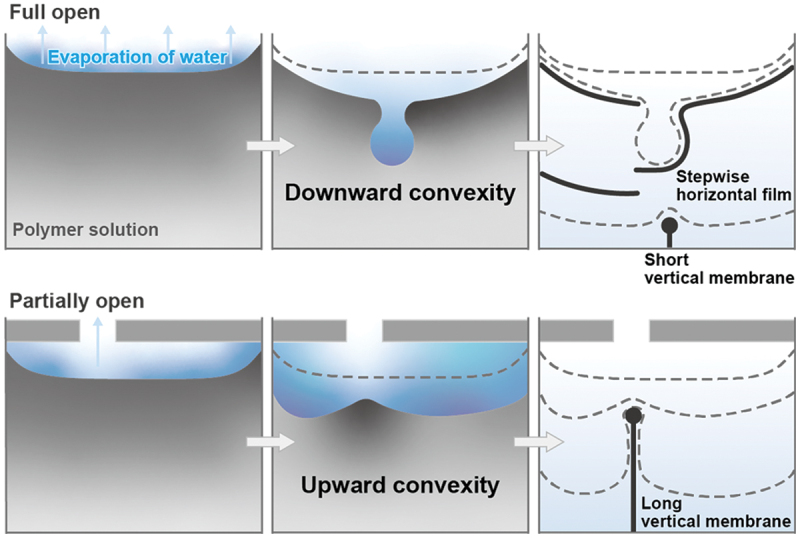
The strategic design of aperture configurations enables the regulation of interfacial convexity through localized humidity modulation. In a fully open cell, accelerated solvent evaporation from the polymer solution induces downward interfacial deformation followed by vertical membrane growth. In contrast, a partially open cell maintains elevated local moisture levels to suppress solvent evaporation and promote vertical membrane formation from the start due to upward interface deformation.

In this study, we designed an open system for the meniscus splitting of an aqueous PVA solution by controlling the vapor phase space for the influx/efflux speed of air containing water vapor. This space control caused substantial local humidity just above the evaporative interface. An aqueous PVA solution was used as the liquid phase to demonstrate its versatility and universality in open systems. As shown in Figure 1, two types of interfacial deformation and characteristic polymer deposition can be controlled by adjusting the humidity through changing the aperture design. Different from polysaccharides forming vertical membranes by splitting the evaporative interface [27], PVA deposition occurs along the horizontal direction when the top of the cell is fully open. This would be because the dehydration of PVA happens much more immediately and bridges the gap on a wide area of the evaporative interface. The rapid evaporation of water from the interface in contact with dry air causes downward convex interface deformation near the center of the cell. As horizontal film formation maintains high humidity, polymer deposition contributes to membrane growth in the vertical direction. In contrast, a partially open aperture suppresses water evaporation and maintains high humidity during the initial drying process. This condition induces the evaporative interface to deform with an upward convexity, and polymer deposition results in vertical membrane formation, similarly seen in polysaccharide solutions in previous works. To test this hypothesis, we investigated the fluid system by focusing on the fundamental properties of the polymer solution, such as its viscosity and the size and position of the apertures.

## Results and discussion

**Figure 2.**
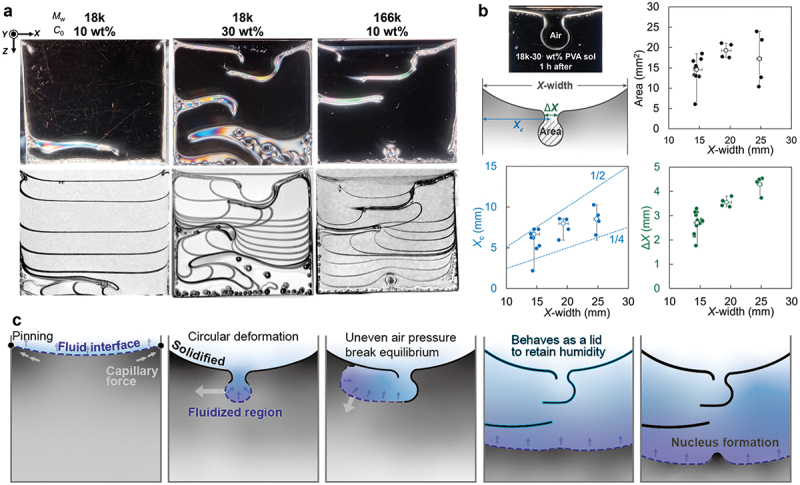
Interfacial shape changes into downward convex and polymer deposition in the horizontal direction in a fully open aperture cell. (a) Images of drying interface (upper) and superimposition of the time evolution (lower) of PVA solutions with different molecular weights (*M*_w_) and initial concentrations (*C*_0_). The number of images for superpositions: 10 during 30 h (18k, 10 wt%), 20 during 65 h (18k, 30 wt%), 15 during 60 h (166k, 10 wt%); cell size: 25 mm-width, 1 mm-gap, ~20 mm-depth; drying temperature: 60 °C; relative humidity outside of the cell: ~5%. (b) Geometric analysis of the air hole during the down-convex interfacial change of the 18k-30 wt% PVA solution in different cells, *X*-width, 1 mm-gap, ~20 mm-depth, where *X* = ~15, ~20, and ~25 mm. (c) Schematic of the interface shape transition in the 166k-10 wt% PVA solution.

Two different molecular weight PVA (18 and 166 kg/mol) aqueous solutions were dried from a limited space under constant temperature and controlled relative humidity (60 °C and ~ 5% RH), based on previous studies (Supporting Information, Figure S1) [24]. Figure 2a and Supporting Information, Movie S1 show the drying process depending on the molecular weight and initial concentration. For the low molecular weight and low initial concentration case (18k-10 wt%), there was no change in the shape of the interface until just before the end of drying. After a monotonous descent, the interface moved horizontally, forming a horizontal film parallel to the *X* direction on the bottom of the cell. In contrast, at a higher initial concentration (18k-30 wt%), the interfacial behavior in the early drying stage became complex. Hole-like sinking of the interface occurred at the beginning of the drying process. This air hole extended in the *X* direction while maintaining its curvature. This is due to the capillary forces, which is strongly affected by the surface tension. When it contacted the sidewall, it sank slightly in the *Z* direction and extended in the opposite *X* direction. The sinking and horizontal deformation of the interface were repeated, resulting in the formation of several horizontal films.

The air holes formed in the initial phase were reproducible regardless of the cell *X*-width. The formation of air holes and their subsequent horizontal deformation can be attributed to the interplay of capillary forces, solvent evaporation rates, and the inherent properties of the PVA solution. Figure 2b shows the results of the geometric analysis of the air holes. The interface shape of the hole-like sinks observed 1 h after the start of drying for the 18k-30 wt% sample was measured. The area of the circular sink is determined by approximating a regular circle, *X*_c_ is the distance from the sidewall to the center of the circle, and Δ*X* is the width of the interface where the sinking occurred. These values were plotted against the *X*-width of the cell. The area of the air holes varied regardless of the *X-*width; however, the maximum value increased proportionally. The *X*_c_ values, which indicate the location of the holes, are distributed between half and quarter of the cell width, indicated by the auxiliary lines in the graph. Furthermore, Δ*X* increased in proportion to the increase in *X*-width, consistently equating approximately one-fifth of the *X*-width. From these geometric analyses, the formation of air holes was observed for all cell sizes, but the hole location and width depended on the *X*-width of the cell, which suggests that the convection of air in the limited space is a determining factor. Although the width of the sink is affected by the cell width, the area of the hole is independent, indicating that the circular sink region remains fluid and does not dry. Owing to the instability of this region, it is assumed to be a limit on its size and ability to expand horizontally along the top of the solidified horizontal films.

In the higher molecular weight 166k-10 wt% PVA case, stepwise horizontal films and a short vertical membrane were formed (see Figure 2a). Interfacial behavior similar to that of the 18k-30 wt% PVA solution was also observed. After the horizontal deformation of the interface, it became a meniscus, resembling the initial state, and formed a vertical membrane. The solidification process of the horizontal film and the formation of the nucleus as the origin of the vertical membrane were considered to proceed as shown in Figure 2c. Initially, the entire area at the evaporation interface remained fluid, and the contact area with the sidewall was pinned. The solute concentration near the sidewall increased because of capillary flow, leading to solidification on the surface in contact with dry air. The effect of capillary flow was observed from the sidewall to a quarter of the cell width, and the solidified area extended to the inside of the cell. The interface that maintained fluidity near the center of the cell width was deformed to reduce the volume of the liquid phase. At this point, the sinking of the interface was observed as the formation of a new concave meniscus with a different curvature. This depression was uniform until it was pressurized to the point where the Laplace pressure became zero, when it deformed into a circular shape. However, uneven atmospheric pressure is caused by convection of the gas phase and differences in humidity owing to differences in evaporation rates, with the solidification area acting as a barrier, thus causing the interface to expand horizontally. When a stepwise horizontal film is formed by the deformation front propagating horizontally, it behaves as a lid to maintain a high humidity at the interface, thus inhibiting solidification. In this state, nucleation occurs via self-organization through polymer entanglement.

The measurement of physical parameters revealed a difference in the interface shape transition, which depended on the initial concentration of the aqueous solution and the molecular weight of PVA. The properties of PVA depend on the molecular weight, which indicates the degree of polymerization, and also on the degree of saponification, which indicates the number of hydroxy groups. In solution, the number of inter- and intramolecular hydrogen bonds due to these differences affects the properties [37–44]. The physical properties of the solution at different concentrations and surface energy measurements showed differences in the viscosity gradient depending on the molecular weight and polymer concentration (Supporting Information, Figure S2). The changes in viscosity due to differences in molecular weight can be explained using the Mark – Houwink – Sakurada equation $\left[\eta \right]=KM_{w}^{\alpha }$ for the relationship between intrinsic viscosity [η] and molecular weight *M*_w_ [45,46]. The intrinsic viscosity was calculated to be 40 and 122 mL/g for an average molecular weight of 18 and 166 kg/mol, respectively ($\alpha =0.50,K=300\times 10^{-3}\mathrm{mL}/g$ [47]). This theoretical value is consistent with the intrinsic viscosity calculated from the viscosity measured by EMS (30 and 123 mL/g for molecular weights of 18 and 166 kg/mol, respectively) using Martin’s equation $\ln\eta_{\mathrm{sp}}/C=\ln\left[\eta \right]+K_{M}\left[\eta \right]C$, where η_*sp*_ is the specific viscosity, *C* is the weight-based concentration, and *K*_*M*_ is the Martin parameter [48].

Viscosity increased in an exponential-like manner with concentration, as observed in the semi-logarithmic plot (see Figure S2A). The rate of this increase varied depending on the molecular weight. This trend is also supported by theoretical predictions based on Martin’s equation, which accounts for the intrinsic chain properties and interchain interactions by incorporating both linear and exponential components (Supporting Information, Figure S3A). From the viscosity – concentration master curve [49–53], two critical points, C* and C**, were obtained at which the slope changed (Supporting Information, Figure S3B). As the molecular weight increases and the molecular chain becomes longer, the critical concentration C* decreases, and the viscosity changes more drastically as the concentration changes. The critical concentration C** at which the entanglement started was confirmed only for 166k PVA, suggesting that 18k PVA maintains a semi-dilute region, even at high concentrations. Therefore, the nucleation to form a vertical membrane requires a specific viscosity region.

**Figure 3.**
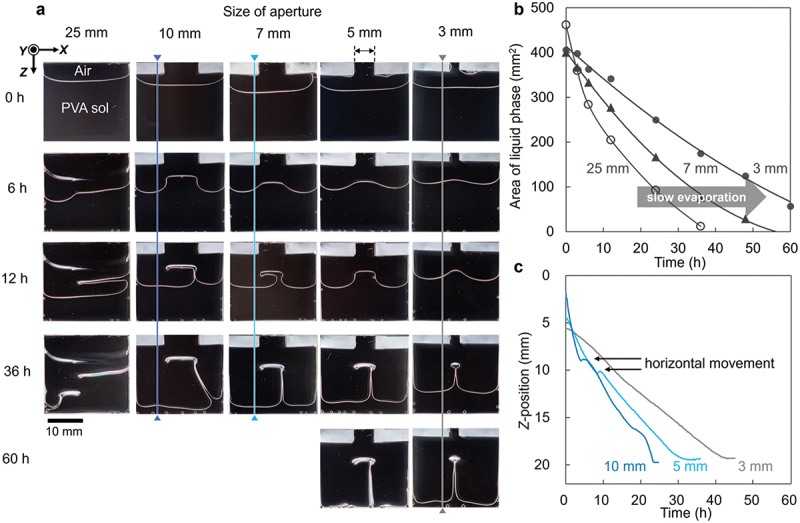
Open system for water evaporation and specific polymer deposition. (a) Time course images of polymer deposition from cells with various aperture sizes. (b) Decrease in the liquid area in the XZ plane for different given aperture sizes. (c) Displacement velocity of the interface at the positions depicted in a. Initial solution concentration: 10 wt% PVA (*M*_w_ ~166k); cell size: 25 mm-width, 1 mm-gap, ~20 mm-depth; drying temperature: 60 °C; relative humidity outside of cells: ~5%..

Vertical membrane formation occurred when the viscosity was appropriate, humidity was high, and the interface remained fluid (see Figure 2c). Based on this consideration, different drying conditions were tested using cells with pre-attached lids to maintain the internal humidity. We designed a cell with a lid on part of its aperture. Using this method, the evaporation rate was controlled, and the humidity at the evaporation interface was maintained, resulting in a long vertical membrane. By tuning the size of the aperture of the cell, horizontal polymer deposition was suppressed, and a vertical membrane was formed just below the aperture, showing meniscus splitting (Figure 3a). The trend was also observed for PVA with a high saponification degree (Supporting Information, Figure S4). During drying, the decrease in the liquid area depended on the size of the aperture (Figure 3b). This suggested that a smaller aperture maintained more humidity at the evaporation interface, which suppressed the drastic increase in viscosity at the interface. Humidity retention is not affected by external humidity and is due to effective aperture design (Supporting Information, Figure S5). The displacement velocity at the interface was measured using the same method as in previous studies [35], which depends on the shape of the nucleus (Figure 3c). For an aperture of 3 mm, the descending speed of the interface exhibited monotonicity. In contrast, larger apertures, such as those of 5 or 10 mm, showed plateau states, indicative of the horizontal deformation of the interface. When the interface does not descend monotonically and a plateau region appears, a ‘Γ-shaped membrane’ with an oblong circle of nuclei is formed. When the aperture size was further reduced, the nucleus changed to a point shape. The minimum aperture size for nucleation was approximately 0.8 mm, and vertical membrane formation was not observed when the aperture size was smaller than this (Supporting Information, Figure S6). The pre-attached lid above the cell prevented solidification from the contact line with the sidewall by maintaining the humidity. However, if the aperture is too large, solidification occurs at the center of the cell in contact with the outside dry air, resulting in an oblong circular nucleus.

**Figure 4.**
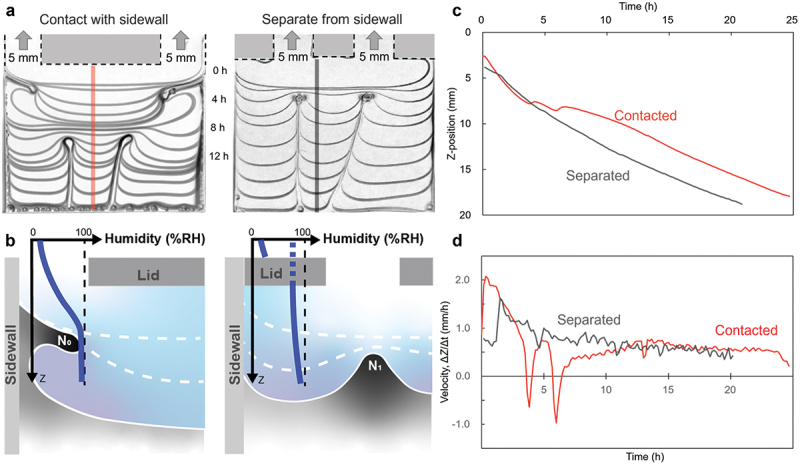
Role of the airflow pathway in the air phase. (a) Superimposition of the time evolution of interface shapes for two apertures in contact with (left) and separated from the sidewalls (right). (b) Schematic of humidity distribution in the area 5 mm from the sidewall with the airflow pathway along/not along the sidewall. (c) Time course of the interface in the Z direction at the position depicted in a., close to the center of the cell. (d) Time course of evaporation velocity for each aperture shape. Initial solution concentration: 10 wt% PVA (*M*_w_ ~166k); cell size: 25 mm-width, 1 mm-gap, ~20 mm-depth; drying temperature: 60 °C; relative humidity outside of cells: ~5%.

In the case of a single aperture, one vertical membrane was formed with the nucleus length corresponding to the size of the aperture. To investigate how membrane formation changed, the number of apertures was increased. Using a cell with two apertures, membrane formation was compared when the apertures were separated from and in contact with the sidewalls (Supporting Information, Movie S2). Vertical membranes were formed after the horizontal interfacial displacement when the apertures and sidewalls were in contact (Figure 4a, left). In contrast, when the apertures were separated from the sidewalls, vertical membranes formed directly below the apertures (Figure 4a, right). These different interface behaviors during the initial drying process can be explained by humidity changes around the corners of the cells. The formation of the horizontal film and its starting point N_0_ is suggested to be due to the air – liquid – solid three-phase contact line being exposed to air and fixed during drying by the solidification of PVA (Figure 4b, left). The three-phase contact line is the site where accumulation by capillary force is the strongly affects, and the contact line is pinned immediately. During drying, the three-phase contact lines are fixed because of the constant evaporation caused by the rapid decrease in relative humidity. As shown in Figure 4b, right, N_0_ formation is suppressed, and the starting point of vertical membrane formation, N_1_, occurs from the initial drying stage. This is because the three-phase contact lines were not exposed, and the relative humidity was maintained at a high value.

The *Z* position and velocity of the descent of the interface are shown in Figure 4c and d, respectively. When the apertures are separated from the sidewalls, the position of the interface decreased continuously and monotonically. In contrast, when the apertures are in contact with the sidewalls, two plateau periods are observed, which were caused by the horizontal deformation of the interface. The plateau period exhibited a negative velocity value, indicating that the interface in the central region increased slightly and was attracted by the horizontal deformation of the interface near the sidewall. This clearly suggested that the interface in the central region is still fluid, and no film has formed yet. A comparison of the two plateau periods revealed a large deceleration in the second plateau region involved in the formation of the right horizontal membrane, which was caused by polymer deposition and film formation in the horizontal direction. As the number of apertures was further increased, the interfacial deformation and membrane formation became more complex, and vertical membranes did not form according to the number of apertures (Supporting Information, Figure S7). This was due to the proximity of the apertures, which reduced the individual functions of the apertures. These observations emphasize the complexity of membrane formation, which is influenced by the aperture width and position and affects the local humidity. Thus, the control of polymer deposition by the exterior environment allows for vertical membrane formation and meniscus splitting.

## Conclusion

The deformation of the evaporative interface in the meniscus splitting phenomenon was investigated toward the universal design for the dry/wet non-equilibrium conditions in an open system. Controlled vertical membrane formation through meniscus splitting was successfully demonstrated with aqueous PVA systems using a cell aperture design. The geometric design of the aperture, including its size and position, directly affects the humidity gradient and interface deformation. In an open system with apertures, an initial meniscus with downward convexity was capable of transitioning into a meniscus with multiple upward convexities. This deformation was necessary for the splitting phenomena. The demonstration with PVA elucidated two types of transitions: downward-to-downward and downward-to-upward. Owing to the easy drying properties of PVA, controlling the initial viscosity and local humidity are important factors.Moreover, the aperture design allows the number of membranes to grow in the vertical direction. Thus, extending the study to a wider range of materials might reveal that meniscus splitting is independent of the chemical species, indicating that the underlying mechanism is governed by physical interactions. The successful validation of this behavior in a challenging system such as PVA provides a foundation for verifying its universality across a wide range of systems. This capacity for humidity-mediated control of interfacial deformation has profound implications not only for phenomenology but also for the creation of self-organizing materials [54–56]. We believe that its applicability extends to smart materials having oriented structures with controlled directional responses to various stimuli such as vapor [26], cations [34], and pH [36].

## Supplementary Material

Supplemental Material

Supplemental Material

Supplemental Material

## Data Availability

The authors declare that all the data supporting the findings of this study are available in the Article and Supplementary Information files.
