# Supplementary material for: Design of open systems for meniscus splitting demonstrated using an aqueous polymer solution
Source: Sci Technol Adv Mater. 2025 May 28;26(1):2512704. doi: 10.1080/14686996.2025.2512704 (PMC12180327; doi:10.1080/14686996.2025.2512704)
Supplement: Supplemental Material [file TSTA_A_2512704_SM4375.docx]

Supplementary Information

**Design of open systems for meniscus splitting**

**using an aqueous polymer solution**

Reina Hagiwara^1^, Kosuke Okeyoshi^1*^

^1^Graduate School of Advanced Science and Technology, Japan Advanced Institute of Science and Technology, 1-1 Asahidai, Nomi, Ishikawa 923-1292, Japan

* Correspondence should be addressed.

E-mail: okeyoshi@jaist.ac.jp

**Table of Contents**

1. Experimental methods
2. Characterization of the PVA solution
3. Membrane shape formed by aperture design

**I. Experimental methods**

**Materials**

Polyvinyl alcohol with molecular weights (*M*_w_) of 13—23 (PVA-18k) and 146—186 kg/mol (PVA-166k) (87–89% hydrolysed) were purchased from Sigma-Aldrich. The aqueous PVA solution was prepared by dispersing the polymer powder in pure water, stirring at room temperature (~ 25 °C) for 1 h, heating it in a 90 °C water bath for 2 h, then stirring at room temperature for 1 h. The solution samples were stored at room temperature.

**Drying experiment**

Drying experiments were performed by injecting the polymer solution into a top-open cell with a narrow gap, as shown in **Fig. S1A**. The Hele–Shaw cell consisted of two glass slides (S1111, Matsunami Glass Ind. Ltd.) and a polyvinyl chloride (PVC) sheet that is inert under thermal conditions during drying, and easy to prepare customized-sized cells. Whereas a small amount of bubbles are generated from the spacer during the long heating, the amount is much smaller than in other materials such as silicone rubber. The open system was designed by changing the shape of the PVC sheet. The samples were placed in an oven (VOS-210C, EYELA, Japan) with an air circulator maintained at constant temperature under atmospheric pressure. In the drying experiments for the spatiotemporal analysis, the samples were placed in an observation box (**Fig. S1B**). The temperature and humidity of the observation box were controlled using a heat pen connected to a circulation tube and a rubber heater on the inner wall of the box. Dry air from the pen heater flowed into the observation box as shown in **Fig. S1C**, keeping the temperature and humidity constant (**Fig. S1D**). Interface changes during drying were monitored using two orthogonally assembled polarizers and a first-order retardation plate (λ = 530 nm), and images were taken at 10-minute intervals.


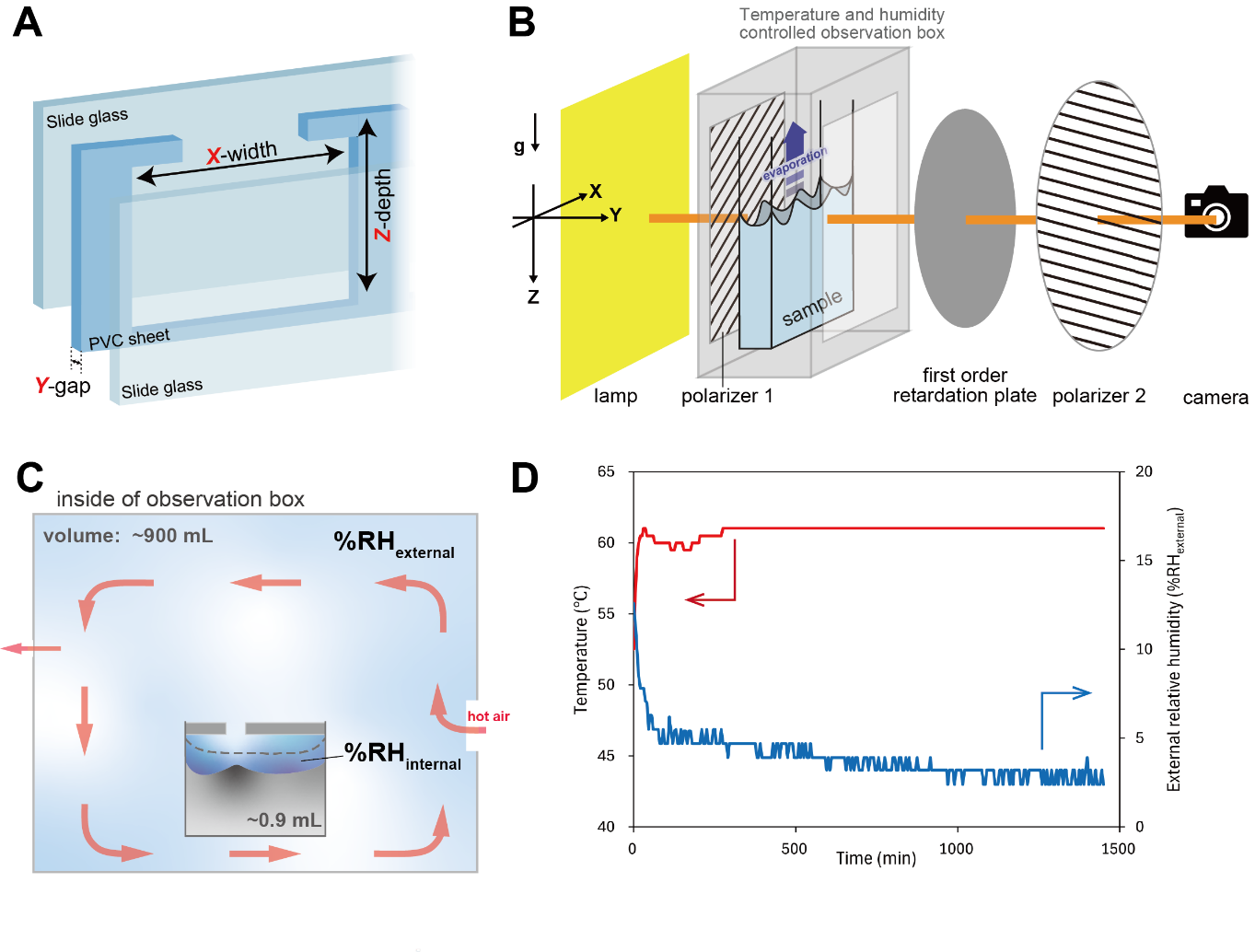


**Fig. S1** Schematic of the experimental setup. **A.** Cell details. The size of the cell is indicated by the *X*-width, *Y*-gap, and *Z*-depth. **B.** Observation box installation conditions and environment. **C.** Dry air flow in the observation box. **D.** Time variation of temperature and humidity in the observation box.

**II.** **Characterization of the PVA solution**

The viscosity (*η*) and density (*ρ*) of the solutions were measured over a wide concentration range for each molecular weight PVA solution (**Fig. S2A** and **B**). The viscosity was measured using a non-contact electromagnetic spinning method using an EMS-1000S (Kyoto Electronics Manufacturing Co., Ltd., Japan), and the density was measured using an oscillatory method based on the U-tube oscillation principle with a DMA 4501 (Anton Paar, Austria). The contact angle (*θ*) and surface tension (γ) were measured using a DMs-401 instrument (Kyowa Interface Science Co., Ltd., Japan) (**Fig. S2C**). The capillary length *l* was calculated using Equation 1, where *g* is the acceleration owing to gravity (9.8 m/s^2^).

$$l=\sqrt{\gamma/{\rho g}} (1)$$

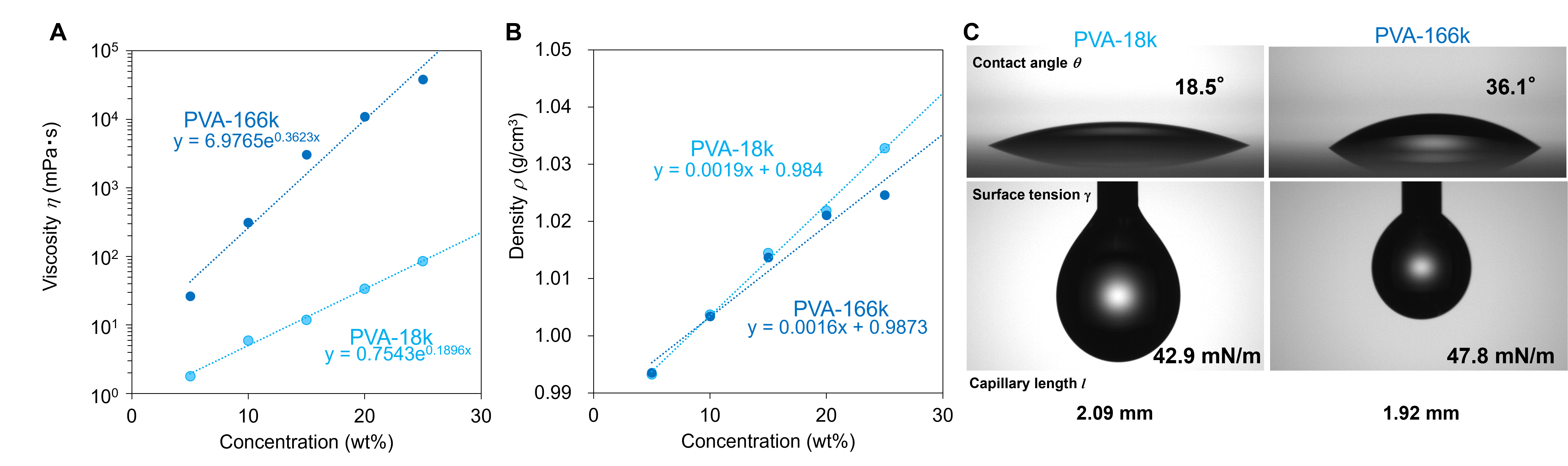


**Fig. S2** Physical properties of PVA at different molecular weights. **A.** Viscosity of each aqueous polymer solution. **B.** Density of each aqueous polymer solution. **C.** Difference in surface energy. Contact angle on glass (upper), surface tension (middle), and capillary length (bottom).

Among the five datasets, the viscosity and contact angle showed remarkable differences depending on the molecular weight of PVA. Although the contact angle changed twice, the effect was considered small because both angles were low. Major changes appeared in both the intrinsic viscosity and the rate of increase in viscosity. **Fig. S3A** shows a graph of $\ln{\eta_{sp}}/C$ as a function of concentration. The intrinsic viscosity is calculated from the slope of this graph based on Martin’s equation (Equation 2). Specific viscosity *η*_sp_ is given by $(\eta/\eta_{0})-1$, where *η* is the measured value and *η*_0_ is the viscosity of water (0.47 mPa･s). The calculated intrinsic viscosity was similar to the theoretical value (40 and 122 mL/g of 18 and 166 kg/mol, respectively) based on the Mark–Houwink–Sakurada equation: $\left[ \eta\right]=KM_{w}^{\alpha}$. Next, using a log–log plot of *η_sp_* versus the dimensionless coil overlap parameter *C*[*η*] for the slope of the viscosity increase (**Fig. S3B**), three regions corresponding to the dilute, semidilute, and concentrated domains were identifiable. Within each domain, the *η_sp_*–*C*[*η*] relationship is described by a linear dependence, with transitions from one domain to another accompanied by a change in the slope, as indicated by the critical concentrations *C** and *C***. For PVA-18k, the slopes are 1.2 and 2.5, and the critical concentration *C** resulting from the initial point of contact between individual chains was 4.7 wt%. In contrast, for PVA-166k, the slopes were 1.5, 3.3, and 4.8, with a *C** of 2.5 wt% and a *C*** of 6.3 wt% as the initial point of the entanglement of the polymer chains.

**
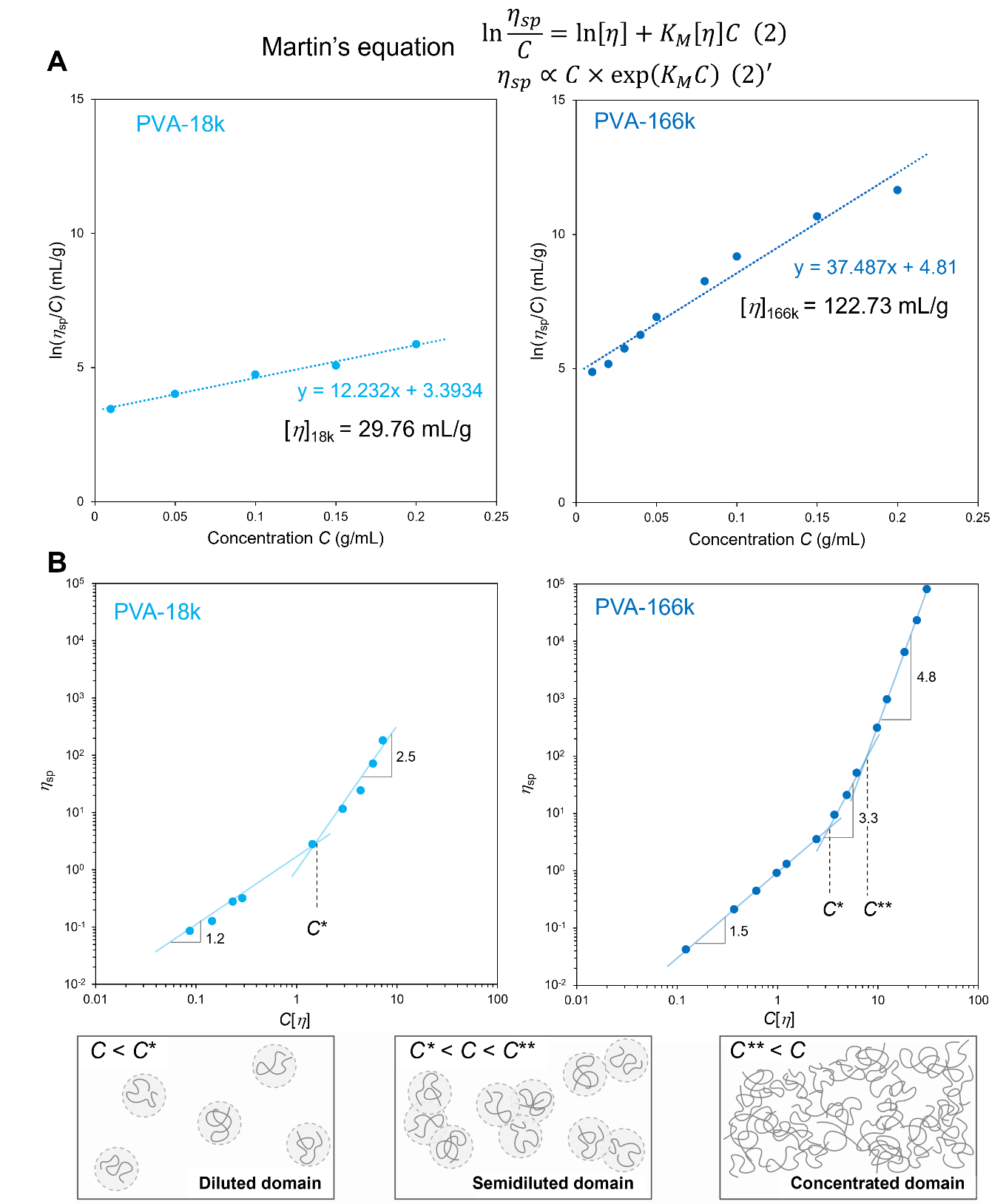
**

**Fig. S3** The equation for viscosity gradient as a function of polymer concentration. **A.** Calculation of intrinsic viscosity using Martin’s equation. **B.** Log–log plots of *η*_sp_ versus *C*[*η*] of PVA with a molecular weight of 13—23 (left) and 146—186 (right)kg/mol and the corresponding models of chain aggregation for each.

**III. Membrane shape formed by aperture design**

The drying results for higher saponification degree (~100%) PVA solutions are shown in **Fig. S4**. The PVA with a higher saponification degree results in lower solubility, and the aqueous solution shows higher viscosity. The main materials used in this study had a saponification degree of 88% and a molecular weight of 166k. A comparison was made using initial viscosity about half or twice as high as it, and almost the same membrane formation was observed just below the initial interface. The similarity in membrane shape indicates that these samples are concentrated regions with entanglement above *C***, and there is no noticeable difference in dehydration characteristics by saponification degree.

**
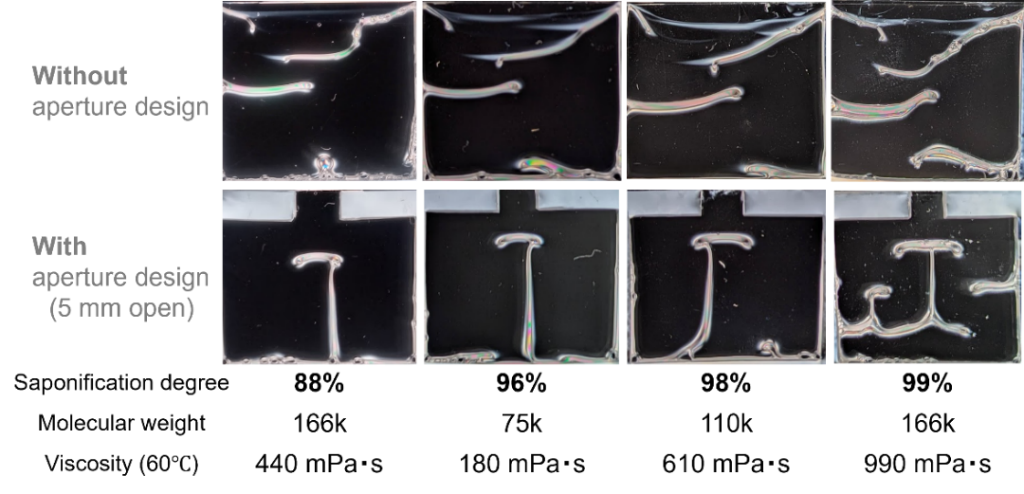
**

**Fig. S4** Membrane shape for each saponification degree. Initial solution concentration: 10 wt% PVA; cell size: 25 mm-width, 1 mm-gap, ~20 mm-depth; drying temperature: 60 °C; relative humidity outside of cells: ~5%.

At lower drying temperatures and higher external humidity (40℃, ~10%RH), similarly shaped membranes were formed in the aperture-design cells (**Fig. S5**). This indicates that the internal humidity near the evaporation interface is more important than the external humidity.

**
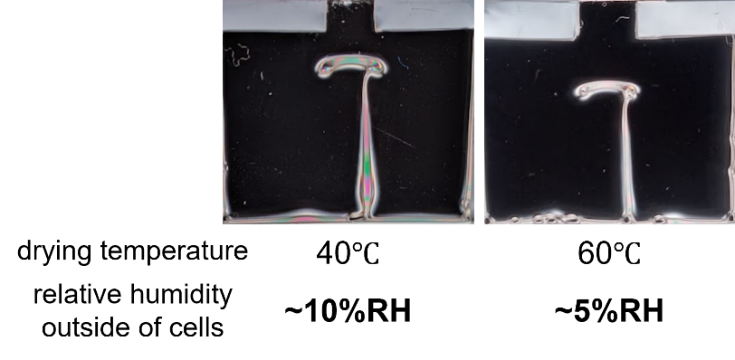
**

**Fig. S5** Membrane formation under different conditions outside the cell. Initial solution concentration: 10 wt% PVA; cell size: 25 mm-width, 1 mm-gap, ~20 mm-depth, aperture size ~5 mm.

When the aperture size was reduced from the full aperture size (25 mm), similar vertical membrane formation was observed in the aperture size range of 1–10 mm. As shown in **Fig. S6**, a vertical membrane was not observed when the aperture size was smaller than 0.8 mm. This is because the interfacial fluctuations cannot grow nonlinearly with the observed time at this drying temperature and humidity condition of the evaporation interface. Therefore, it is possible to induce nucleation by changing the depth of the cell to increase the observation time

**
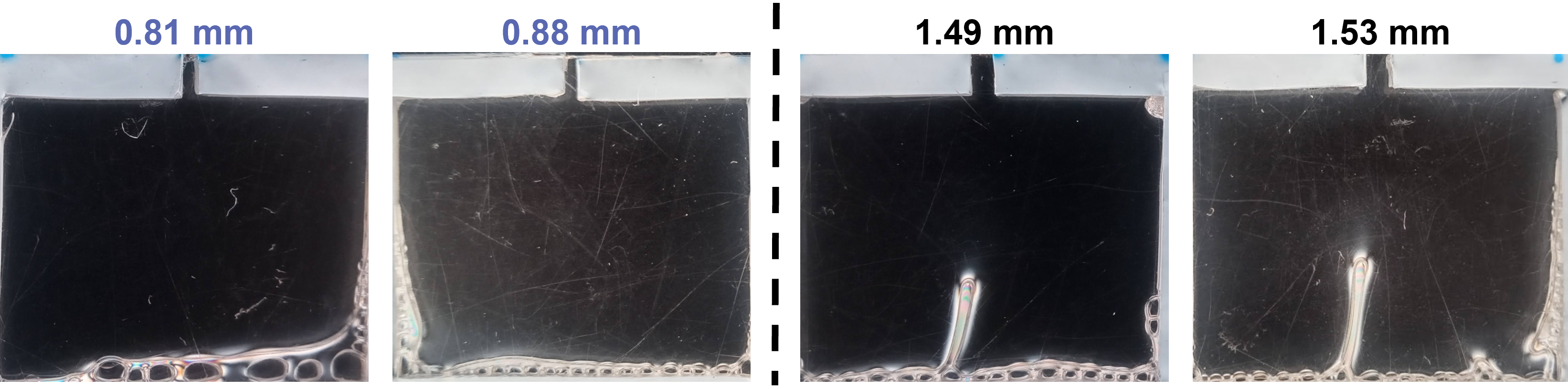
**

**Fig. S6** Minimum aperture length for nucleation. Initial solution concentration: 10 wt% PVA (*M*_w_ ~166k); cell size: 25 mm-width, 1 mm-gap, ~20 mm-depth; drying temperature: 60 °C; relative humidity outside of cells: ~5%.

**Fig. S7** shows the results of the drying experiments in a cell with three apertures. Vertical membranes were not formed according to the number of apertures, either when the apertures at both ends were in contact with or separated from the sidewalls. It is possible that the individual functions of the apertures were reduced because of their proximity to each other. When the aperture was in contact with the sidewall, a starting point of horizontal film N_0_ formed initially, as in the cell with two apertures; however, the stepwise horizontal film formation continued. Even when the apertures were far from the sidewalls, a N_0_ was formed because the distance between the apertures and sidewalls was so small that humidity control near the sidewalls was ineffective.

**
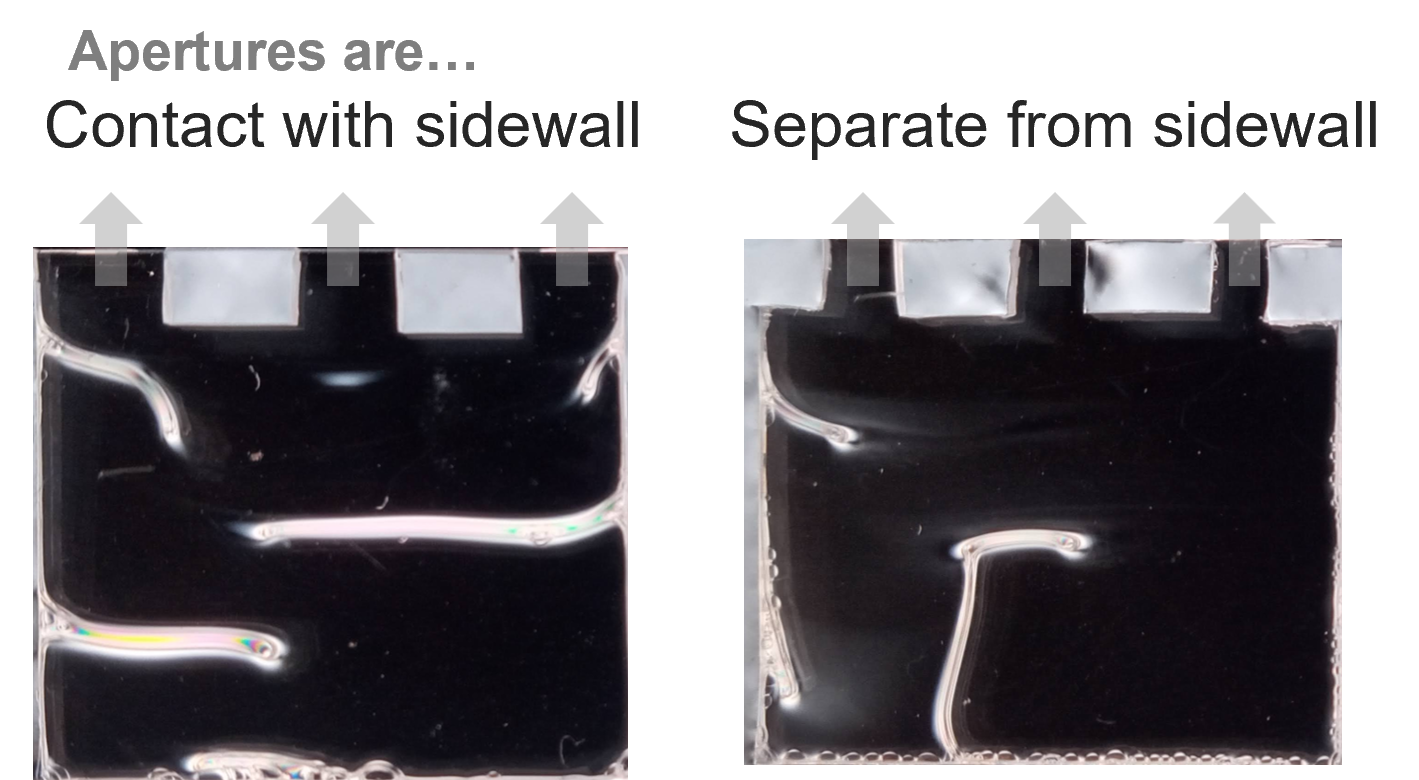
**

**Fig. S7** The complex membrane formation in a cell with three apertures. Initial solution concentration: 10 wt% PVA (*M*_w_ ~166k); cell size: 25 mm-width, 1 mm-gap, ~20 mm-depth; drying temperature: 60 °C; relative humidity outside of cells: ~5%.
